# THE RELATIONSHIPS BETWEEN RELIGIOUS SERVICE ATTENDANCE, SPIRITUALITY, LONELINESS, AND SOCIAL ISOLATION

**DOI:** 10.1093/geroni/igae098.0632

**Published:** 2024-12-31

**Authors:** Harry Taylor, Ann Nguyen

**Affiliations:** University of Toronto, Toronto, Ontario, Canada; Case Western Reserve University, Cleveland, Ohio, United States

## Abstract

The objective of our study is to determine if religious service attendance and spirituality had an association with loneliness and social isolation among older adults. We used the Health and Retirement Study to address our research objective. Religious service attendance was measured dichotomously (no religious service attendance vs. attending religious services at least once a year). Spirituality was measured by Fetzer Institute 4-item Brief Multidimensional Measure of Religiousness/Spirituality. Loneliness and social isolation were operationalized using the UCLA 3-item loneliness scale and by a social network index, respectively. Covariates included age, gender, race, income, employment status, and education. We used multivariable linear regression to examine the relationships between religious service attendance, spirituality, loneliness, and social isolation. We found respondents who did not attend religious services were more likely to be socially isolated. Additionally, spirituality did not have a significant association with social isolation. Respondents who did not attend religious services had significantly greater loneliness in comparison to those who did attend religious services. Furthermore, respondents with higher spirituality scores had lower loneliness scores. We found religious service attendance had an association with social isolation only, while both attendance and spirituality had an association with loneliness among older adults; therefore, improving access to religious institutions and enhancing one’s spirituality may serve as important factors for mitigating social isolation and loneliness among older adults.

